# Post–COVID-19 Condition After SARS-CoV-2 Infections During the Omicron Surge vs the Delta, Alpha, and Wild Type Periods in Stockholm, Sweden

**DOI:** 10.1093/infdis/jiad382

**Published:** 2023-09-04

**Authors:** Pontus Hedberg, Pontus Nauclér

**Affiliations:** Department of Medicine Huddinge; Division of Infectious Diseases, Department of Medicine Solna, Karolinska Institutet; Department of Infectious Diseases, Karolinska University Hospital, Stockholm, Sweden

**Keywords:** COVID-19, long COVID, post–COVID-19 condition, SARS-CoV-2

## Abstract

Little is known about the post–COVID-19 condition (PCC) after infections with different SARS-CoV-2 variants. We investigated the risk of PCC diagnosis after primary omicron infections as compared with preceding variants in population-based cohorts in Stockholm, Sweden. When compared with omicron (n = 215 279, 0.2% receiving a PCC diagnosis), the adjusted hazard ratio (95% CI) was 3.26 (2.80–3.80) for delta (n = 52 182, 0.5% PCC diagnosis), 5.33 (4.73–5.99) for alpha (n = 97 978, 1.0% PCC diagnosis), and 6.31 (5.64–7.06) for the wild type (n = 107 920, 1.3% PCC diagnosis). These findings were consistent across all subgroup analyses except among those treated in the intensive care unit.

## Teaser

This population-based cohort study revealed a reduced risk of getting diagnosed with the post–COVID-19 condition after a primary infection during the omicron surge as compared with the delta, alpha, and wild type periods.

Besides the substantial mortality and morbidity attributed to coronavirus disease 2019 (COVID-19), an array of postacute and long-term health problems have been described, often referred to as “long COVID” or the “post–COVID-19 condition” (PCC) [1, 2]. While several previous studies have demonstrated a reduced risk of severe COVID-19 for infections caused by the omicron variant as compared with the delta and alpha variants, less is known about the risk of developing PCC [3, 4]. A UK study of self-reported data from a mobile application found omicron cases to be less likely to feature PCC when compared with delta cases [5]. Similarly, a Norwegian cohort study revealed a reduced risk of post–COVID-19 complaints from ≥90 days after a positive test result for omicron vs delta [6]. Reasons for potential risks of developing PCC across SARS-CoV-2 variants might be multifactorial, including differences in lower respiratory tract involvement and in the proportion with natural and vaccine-induced immunities [7, 8]. We previously showed that an *ICD-10* diagnosis of PCC has a high correlation with excess health care usage in patients with SARS-CoV-2 infections [9]. In this study, we aimed to investigate the occurrence and risk of PCC after a SARS-CoV-2 primary infection during the omicron surge as compared with the delta, alpha, and wild type periods in Stockholm County, Sweden.

## METHODS

### Study Design, Data Sources, and Study Population

We conducted a retrospective population-based cohort study of PCC in individuals residing in Stockholm County, Sweden. Data were linked from 5 sources: SmiNet (where all positive test results for SARS-CoV-2 are reported), Stockholm Regional Healthcare Data Warehouse, Statistics Sweden, the National Vaccination Register, and the Swedish Intensive Care Registry (Supplementary Text 1). The study population consisted of those born in 2018 or earlier with a polymerase chain reaction–verified SARS-CoV-2 primary infection from 1 October 2020 to 8 February 2022, a period when public testing was available and the PCC diagnosis was used. To ensure capture of baseline characteristics, the study population was restricted to residents who had lived in Stockholm County since 31 January 2019, 1 year before the first confirmed SARS-CoV-2 infection in Sweden. Patients with <90 days of follow-up after the first positive test result were excluded as previously described [9]. An infection was classified as wild type, alpha, delta, or omicron if confirmed through whole genome sequencing or if the positive test result occurred during the variant periods described by the Public Health Agency of Sweden [10].

### Study Outcomes and Other Variables

The study outcome was a PCC diagnosis (*ICD-10* code U09.9) given in primary care, outpatient specialist care, or inpatient care 90 to 240 days after the first positive test result. The time limit of 90 days after the first positive test result was used in accordance with the clinical case definition of PCC from the World Health Organization [1]. The upper limit of 240 days was used to ensure equal follow-up time for all variants, since this was the longest follow-up possible for all omicron infection episodes before the study period ended (15 October 2022). Individuals were censored at the date of death, date of moving out of Stockholm County, or 15 October 2022, whichever occurred first. Other collected variables included age, sex, sociodemography, comorbidities, COVID-19 vaccination status before the infection, and severity of the SARS-CoV-2 primary infection. Descriptions of study variables are available in Supplementary Table 1.

### Statistical Analyses

We first described characteristics of the 4 variant cohorts. The percentage and number receiving a PCC diagnosis was reported for all cohorts as well as 11 predefined subgroups for each cohort. These subgroups were based on characteristics that might be associated with the risk of developing PCC: age, sex, severity of acute SARS-CoV-2 infection, and vaccination status before the infection [9, 11]. The cumulative incidence of PCC was estimated with death and moving out of the region as competing events. Adjusted Cox proportional hazards regression models were used to model the risk of being diagnosed with PCC in the delta, alpha, and wild type cohorts when compared with the omicron cohort. Age, sex, and their interaction, as well as the studied sociodemographic variables and comorbidities, were included in the models. These analyses were done in the overall cohorts and in the predefined subgroups, excluding the variable that defined the subgroup from the models. Education level, income, and sick days were excluded from the models in the subgroup analysis of patients aged 1 to 17 years. For the subgroup analysis of vaccinated individuals, only those aged ≥18 years from the omicron and delta cohorts were included, since they all had the possibility to receive ≥2 doses before the infection. All analyses were conducted with R version 4.1.0.

### Ethics Declaration

The study was approved by the Swedish Ethical Review Board (Dnr 2018/1030-31, COVID-19 research amendments Dnr 2020-01385, 2020-02145, 2020-04069, and 2022-02127-02).

## RESULTS

In the Stockholm County population of 2 262 917, we identified 505 656 infection episodes of which 473 359 primary infections from the same number were included in the subsequent analyses (Supplementary Figure 1): 215 279 in the omicron cohort, 52 182 in the delta cohort, 97 978 in the alpha cohort, and 107 920 in the wild type cohort. Characteristics of these 4 cohorts are presented in Supplementary Table 2.

### Occurrence and Risk of PCC Diagnosis

**Figure 1.**
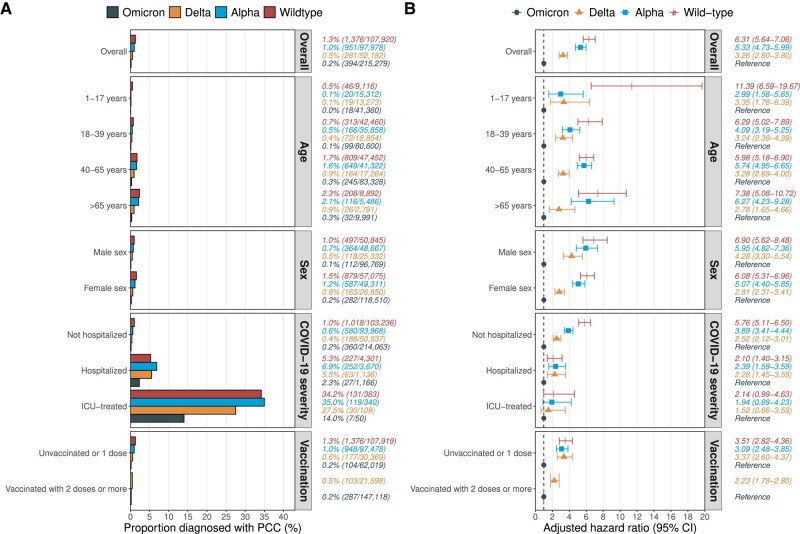
Occurrence and hazard ratio of PCC diagnosis by SARS-CoV-2 variant of concern. *A*, The percentage diagnosed with PCC in the 4 variant cohorts overall as well as the 11 predefined subgroups. Numbers to the right represent the percentage diagnosed with PCC (number diagnosed with PCC / number in cohort). *B*, The adjusted hazard ratio (95% CI) for being diagnosed with PCC in the delta, alpha, and wild type cohorts as compared with the omicron cohort. These analyses were done for the overall cohorts as well as the 11 predefined subgroups. Numbers to the right represent the adjusted hazard ratio (95% CI). Models were adjusted for age (by using restricted cubic splines with 4 knots), sex, age × sex interaction, region of birth, education level, disposable income quartile, days with sickness benefit in 2019, and all studied comorbidities. For the subgroup analyses, the variable that defined the subgroup was excluded from the models (eg, age in the age subgroups). Education level, income, and sick days were excluded from the models in the subgroup analysis of individuals aged 1 to 17 years. For the subgroup analysis of vaccinated individuals, only those aged ≥18 years from the omicron and delta cohorts were included, since they had the possibility to receive ≥2 doses before the infection. ICU, intensive care unit; PCC, post–COVID-19 condition.

In the entire study population, 0.6% (n = 3002) received a PCC diagnosis any time from 90 to 240 days after the first positive SARS-CoV-2 test result. A total of 0.2% (n = 394) in the omicron cohort received such a PCC diagnosis, as compared with 0.5% (n = 281) in the delta cohort, 1.0% (n = 951) in the alpha cohort, and 1.3% (n = 1376) in the wild type cohort (Figure 1*A*). The median time to first PCC diagnosis ranged from 112 days in the omicron cohort to 123 days in the delta cohort (Supplementary Figure 2). The percentage receiving a PCC diagnosis increased with the severity of the acute infection: among individuals treated in the intensive care unit (ICU), 14% (n = 7) in the omicron cohort received a diagnosis, as opposed to 28% (n = 30) in the delta cohort, 35% (n = 119) in the alpha cohort, and 34% (n = 131) in the wild type cohort. When compared with the omicron cohort, the adjusted hazard ratio (95% CI) was 3.26 (2.80–3.80) for the delta cohort, 5.33 (4.73–5.99) for the alpha cohort, and 6.31 (5.64–7.06) for the wild type cohort (Figure 1*B*). Nonomicron variants had a significantly increased hazard of a PCC diagnosis in all subgroup analyses except among those treated in the ICU.

## DISCUSSION

In this population-based cohort study, we observed a reduced risk of getting diagnosed with PCC for individuals with a primary infection during the omicron surge as compared with the delta, alpha, and wild type periods. This was observed in the overall cohorts as well as the 11 subgroup analyses, except among those treated in the ICU, where no significant difference was observed.

Despite methodological differences, our findings of a reduced risk of PCC in the omicron vs delta cohort are in line with the findings from a case-control study in the United Kingdom based on data from a mobile application, reporting 4.5% of the omicron cases and 10.8% of the delta cases to feature PCC [5]. Our findings of a lower occurrence of PCC diagnosis could be due to undiagnosed health-related issues not captured in our study but also that mobile application users are not representative of an entire population. Our results corroborate findings from a Norwegian prospective cohort study where individuals infected with omicron had a lower risk of having any post–COVID-19 complaint >90 days after testing positive as compared with those infected with delta [6]. Potential reasons for a reduced risk of PCC after infections caused by omicron might be differences in the intrinsic properties of different SARS-CoV-2 variants to cause long-term health problems as well as differences in vaccination coverage and population immunity to the SARS-CoV-2 virus [12]. COVID-19 vaccinations and previous infections have been demonstrated to be associated with a lower risk of developing PCC [13, 14]. This could be explained by the reduced risk of developing severe COVID-19, which we previously, as well as in this study, demonstrated to be associated with a substantially higher occurrence of PCC [9]. We are unaware of any studies comparing the risk of PCC in patients with severe COVID-19 across SARS-CoV-2 variants. Although the number treated in the ICU in the omicron cohort was low, possibly leading to insufficient power to detect significant differences, our results highlight the importance of preventing severe COVID-19 to reduce the risk of not only acute morbidity and mortality but also long-term sequelae.

Strengths of our study include the linking of several population-based data sources with high coverage, which enabled inclusion of all polymerase chain reaction–verified SARS-CoV-2 infections and data on sociodemographic variables, comorbidities, and COVID-19 vaccinations, all previously implicated in the risk of getting PCC. Importantly, we included data on primary care diagnoses where most patients with PCC are attended [9]. Furthermore, the study covered a period when the PCC diagnosis was used and public testing was available in Sweden, reducing the risk of differential SARS-CoV-2 testing practices and usage of the PCC diagnosis. Regarding limitations, it is likely that not all individuals experiencing health problems after a SARS-CoV-2 infection seek medical care, thus underestimating the true burden of PCC. However, we do not expect the health care–seeking behavior or access to care to be differentially reduced after the omicron surge.

In conclusion, our results suggest a reduced risk of getting diagnosed with PCC after an omicron primary infection as compared with earlier SARS-CoV-2 variants.

## Supplementary Data

Supplementary materials are available at *The Journal of Infectious Diseases* online. Consisting of data provided by the authors to benefit the reader, the posted materials are not copyedited and are the sole responsibility of the authors, so questions or comments should be addressed to the corresponding author.

## Supplementary Material

jiad382_Supplementary_Data

## Notes

***Author contributions.*** P. N. and P. H. had full access to all the data in the study and take responsibility for the integrity of the data and the accuracy of the data analysis. Both authors read and approved the manuscript. Conceptualization: both authors. Data curation: P. H. Formal analysis: P. H. Funding acquisition: P. N. Investigation: both authors. Methodology: both authors. Project administration: both authors. Resources: P. N. Software: P. H. Supervision: P. N. Validation: both authors. Visualization: P. H. Writing original draft: Both authors. Writing—review and editing: both authors.

***Financial support*.** This work was supported by the Swedish Research Council (Dnr 2021-04809 and Dnr 2021-06540) and the EuCARE Project funded by the European Union’s Horizon Europe Research and Innovation Programme under grant agreement 101046016. The funders had no role in the design and conduct of the study; collection, management, analysis, and interpretation of the data; preparation, review, or approval of the manuscript; and decision to submit the manuscript for publication.

***Data sharing.*** The individual participant data underlying this article were subject to ethical approval and cannot be shared publicly. Data from the deidentified administrative health registry are not freely available due to protection of the personal integrity of the participants.

***Ethics approval and consent to participate.*** The need for consent was waived by the Swedish Ethical Review Authority (Dnr 2018/1030-31, COVID-19 research amendment Dnr 2020-01385), since analyses are based on retrospectively collected data from the administrative health registry.
