# Supplementary material for: Post–COVID-19 Condition After SARS-CoV-2 Infections During the Omicron Surge vs the Delta, Alpha, and Wild Type Periods in Stockholm, Sweden
Source: J Infect Dis. 2023 Sep 4;229(1):133–6. doi: 10.1093/infdis/jiad382 (PMC10786247; doi:10.1093/infdis/jiad382)
Supplement: jiad382_Supplementary_Data [file jiad382_supplementary_data.docx]

Supplementary material for

**Post COVID-19 condition after SARS-CoV-2 infections during the omicron surge compared with the delta, alpha, and wild-type periods in Stockholm, Sweden**

**Authors:** Pontus Hedberg, Pontus Nauclér

Correspondence to [pontus.hedberg@ki.se](mailto:pontus.hedberg@ki.se)

**Table of contents**

| **Content** | **Page** |
| --- | --- |
| Text S1. Descriptions of data sources | 3 |
| Table S1. Descriptions of study variables | 4-7 |
| Figure S1. Study flow chart | 8 |
| Table S2. Characteristics of study population | 9-10 |
| Figure S2. Cumulative incidence of PCC diagnosis, moving out and death in the study cohorts | 11 |
| Supplementary references | 12 |

**Text S1. Descriptions of data sources**

**SmiNet:** SmiNet is the electronic system used for surveillance of communicable diseases in Sweden, owned and operated by PHAS and the communicable disease control units in Sweden [1]. It is used for surveillance of more than 60 notifiable diseases, including COVID-19, according to the Communicable Diseases Act and the Communicable Diseases Ordinance [2,3]. Data on all polymerase chain reaction (PCR) tests positive for severe acute respiratory syndrome coronavirus 2 (SARS-CoV-2) for the study population were used in this study.

**Stockholm regional healthcare data warehouse (VAL):** The Stockholm Regional Council operates the healthcare data warehouse VAL, which contains data from multiple administrative healthcare databases [4]. This data includes information on inpatient stays, outpatient specialist visits and primary care visits (coverage of around 94%) reimbursed by Region Stockholm [5]. Furthermore, the data warehouse contains information on demographics, migration status, nursing home residency, home care services, and collected drug prescriptions. Data on all such characteristics for the study population were used in this study.

**Statistics Sweden:** Statistics Sweden is a governmental agency supplying statistics for decision making, debate, and research [6]. This includes several registers such as the Total Population Register (TPR), and the Integrated Database for Labour Market Research (LISA). Data on region of birth, education level, and disposable family income for the study population were used in this study.

**National Vaccination Register (NVR):** All vaccinations within national vaccination programs and vaccinations against COVID-19 should according to Swedish law be reported to the NVR, which is governed by the Public Health Agency of Sweden (PHAS) [7]. Data on administered COVID-19 vaccine doses for the study population were used in this study.

**Swedish Intensive Care Registry (SIR):** SIR is a national quality register for intensive care which was established in 2001 [8]. SIR prospectively collects data from intensive care unit admission in Sweden, currently including data from all 83 intensive care units in Sweden [9]. Data on all COVID-19 related admissions to an intensive care unit for the study population were used in this study.

**Table S1. Descriptions of study variables**

| **Variable** | **Data sources** | **Missing data** | **Definition** | **Time period** | **Possible values** |
| --- | --- | --- | --- | --- | --- |
| SARS-CoV-2 variant cohort | SmiNet | No | Omicron: First positive test any time from 27 December 2021 to 8 February 2022 or whole genome sequence classified as omicron  Delta: First positive test any time from 28 June 2021 to 26 December 2021 or whole genome sequence classified as delta  Alpha: First positive test any time from 15 February 2021 to 27 June 2021 or whole genome sequence classified as alpha  Wild-type: First positive test any time from 1 October 2020 to 14 February 2021 or whole genome sequence classified as wild-type | First positive SARS-CoV-2 test | Omicron, Delta, Alpha, Wild-type |
| Post COVID-19 condition diagnosis | VAL | No | A PCC diagnosis (ICD-10 code U09.9) given by any healthcare profession in primary care, outpatient specialist care, or inpatient care 90 to 240 days after the first positive test | 90 to 240 days after first positive test | Yes, No |
| Sex | VAL | No | Sex of individual | Birth | Male, Female |
| Age | VAL | No | Age the year of the first positive SARS-CoV-2 test | Year of first positive SARS-CoV-2 test | 1 to 106 years |
| Age category | VAL | No | Age category the year of the first positive SARS-CoV-2 test | Year of first positive SARS-CoV-2 test | 1 to 17 years, 18 to 39 years, 40 to 65 years, >65 years |
| Region of birth | Statistics Sweden | Yes, for 928 individuals | According to the United Nations geoscheme | Birth | Africa, The Americas, Asia or Oceania, Europe, Sweden, Missing |
| Education level | Statistics Sweden | Yes, for 6,578 individuals aged 18 years or above | The highest level of completed education in year 2019 | 2019 | Primary, Secondary, Tertiary, Missing |
| Yearly disposable income quartile | Statistics Sweden | Yes, for 925 individuals aged 18 years or above | The birthyear stratified yearly disposable income quartile in year 2019 | 2019 | Quartile 1, Quartile 2, Quartile 3, Quartile 4, Missing |
| Days with sickness benefit | Statistics Sweden | Yes, for 979 individuals aged 18 years or above | Numbers of net days with sickness benefit in 2019 | 2019 | 0, 1-30, >30, Missing |
| Asthma | VAL | No | ICD-10: J45.X, J46.X | Up to five years before the acute infection | Yes, No |
| Cancer | VAL | No | ICD-10: All codes from C00.X to C97.X besides C44.X, Z51.0, Z51.1  KVÅ: DT107, DT108, DT112, DT116, DT135, DV070, DV071, DV134 | Up to one year before the acute infection  Up to one year before the acute infection | Yes, No |
| Cardiovascular disease | VAL | No | ICD-10: I10.X, I11.X, I12.X, I13.X, I14.X, I15.X (should be registered at least twice during the time period)  ICD-10: I20.X, I21.X, I22.X, I23.X, I24.X, I25.X, I26.X, I27.X, I42.X, I48.X, I50.X, I61.X, I63.X, I64.X | Up to five years before the acute infection  Up to five years before the acute infection | Yes, No |
| Chronic kidney failure | VAL | No | ICD-10: N18.X  ICD-10: Z49.1, Z49.2 (should be registered at least twelve times during the time period)  ICD-10: Z99.2  KVÅ: DR016, DR024 (should be registered at least twelve times during the time period) | Up to five years before the acute infection  One year before the acute infection  One year before the acute infection  One year before the acute infection | Yes, No |
| Chronic liver disease | VAL | No | ICD-10: B18.X, K70.X, K71.7, K72.X, K74.6, K75.X | Up to five years before the acute infection | Yes, No |
| Chronic lung disease (not asthma) | VAL | No | ICD-10: D86.0, D86.2, E84.X, J43.X, J44.X, J46.9, J47.X, J70.3, J84.X, J98.2 | Up to five years before the acute infection | Yes, No |
| Diabetes (type 1 or 2) | VAL | No | ICD-10: E10.X, E11.X | Up to five years before the acute infection | Yes, No |
| Immunocompromised state | VAL | No | ATC: H02AB.X (should be registered at least twice during the time period)  ATC: L01.X  ATC: L04.X  ICD-10: B20.X, B21.X, B22.X, B23.X, B24.X, D57.0, D57.1, D80.X, D81.X, D82.X, D83.X, D84.X, Z94.0, Z94.1, Z94.2, Z94.3, Z94.4, Z94.8  KVÅ: DR04.1, DR04.2, DR04.4, DR04.6, DR04.7  KVÅ: H02AB.X (should be registered at least twice during the time period)  KVÅ: L01.X  KVÅ: L04.X | Up to half a year before the acute infection  Up to one year before the acute infection  Up to half a year before the acute infection  Any time before the acute infection  Up to three years before the acute infection  Up to half a year before the acute infection  Up to one year before the acute infection  Up to half a year before the acute infection | Yes, No |
| Mental health disorder | VAL | No | ICD-10: F20.X-F29.X, F30.X-F39.X, F40.X-F48.X | Up to five years before the acute infection | Yes, No |
| Neurologic disease | VAL | No | ICD-10: F00.X, F01.X, F02.X, F03.X, G10.X, G12.2, G20.X, G30.X, G35.X, G70.X, G71.X, G80.X | Up to five years before the acute infection | Yes, No |
| Obesity | VAL | No | ICD-10: E66.X | Up to five years before the acute infection | Yes, No |
| COVID-19 vaccination status before infection | NVR | No | Number of COVID-19 vaccine doses received any time up until 14 days before the first positive SARS-CoV-2 test | 27 December 2020 up until 14 days before the first positive SARS-CoV-2 test | Unvaccinated, 1 dose, 2 doses, 3 doses |
| Severity of acute SARS-CoV-2 infection | VAL, SIR | No | Hospitalized: A hospital admission with a first positive SARS-CoV-2 test any time from 14 days before admission up until date of discharge and a B34.2, U07.1, U07.2, or U10.9 ICD-10 code as main diagnosis at discharge  ICU-treated: Hospitalized in accordance with the definition above plus admission to the ICU any time during this hospitalization.  Not hospitalized: None of the above | First positive SARS-CoV-2 test any time from 14 days before admission up until date of discharge | Not hospitalized, hospitalized, ICU-treated |

**Abbreviations:** ATC=Anatomical Therapeutic Chemical, COVID-19=Coronavirus disease 2019, ICD-10=International Classification of Diseases 10^th^ revision; ICU=Intensive care unit, KVÅ=Klassifikation av vårdåtgärder (Swedish for classification of healthcare procedures), PCC=Post COVID-19 condition, SARS-CoV-2=Severe acute respiratory syndrome coronavirus 2, SIR=Swedish Intensive Care Registry, VAL= Stockholm regional healthcare data warehouse

**Figure S1. Study flow chart**


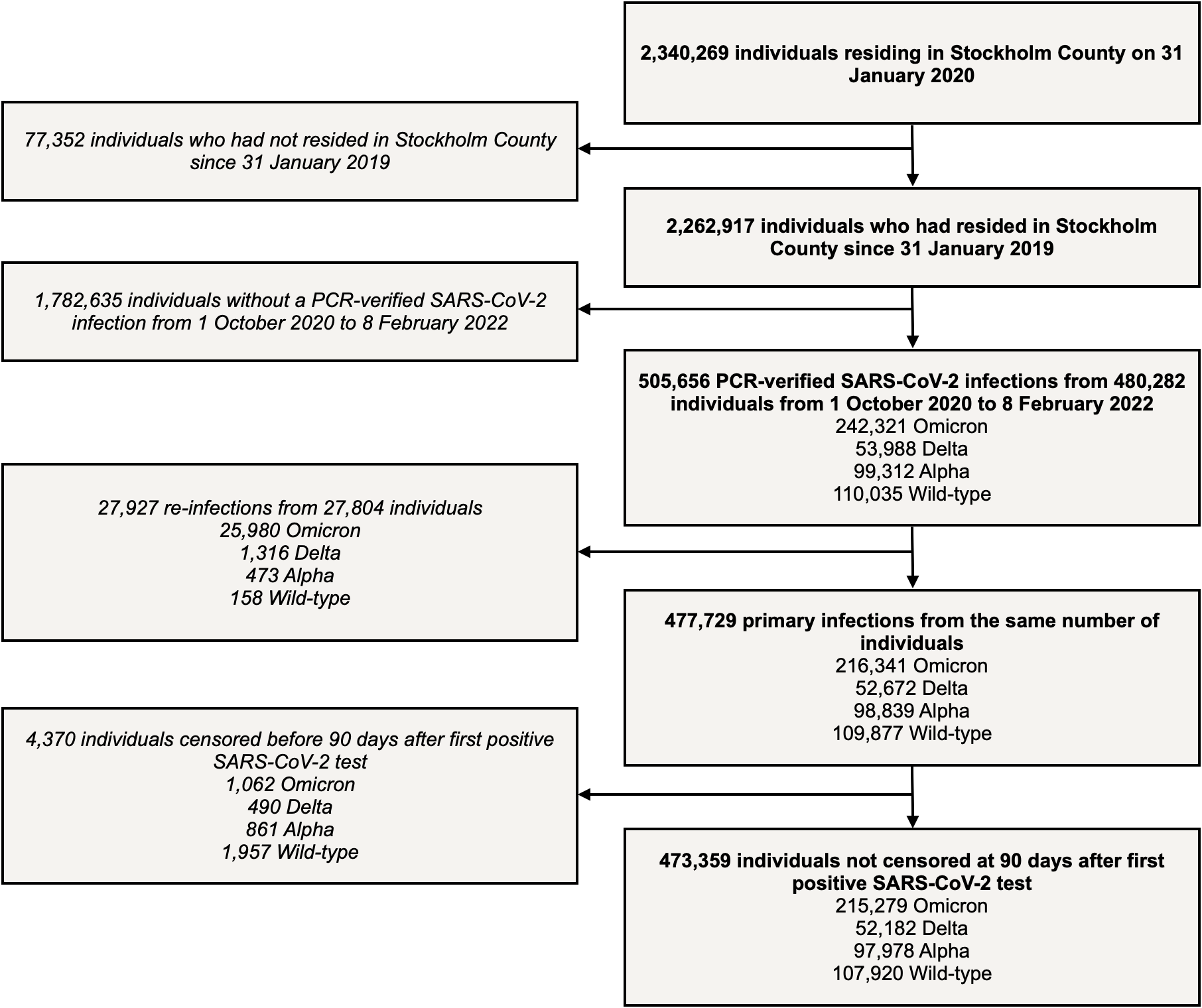


**Abbreviation:** PCR=Polymerase chain reaction, SARS-CoV-2=Severe acute respiratory syndrome coronavirus 2

**Table S2. Characteristics of study population**

| **Variable** | **Omicron (n=215,279)** | **Delta (n=52,182)** | **Alpha (n=97,978)** | **Wild-type (n=107,920)** |
| --- | --- | --- | --- | --- |
| Female sex | 118,510 (55.0) | 26,850 (51.5) | 49,311 (50.3) | 57,075 (52.9) |
| Age, years | 37.0 [23.0, 48.0] | 33.0 [17.0, 47.0] | 38.0 [25.0, 50.0] | 41.0 [28.0, 53.0] |
| 1-17 | 41,360 (19.2) | 13,273 (25.4) | 15,312 (15.6) | 9,116 (8.4) |
| 18-39 | 80,600 (37.4) | 18,854 (36.1) | 35,858 (36.6) | 42,460 (39.3) |
| 40-65 | 83,328 (38.7) | 17,264 (33.1) | 41,322 (42.2) | 47,452 (44.0) |
| >65 | 9,991 (4.6) | 2,791 (5.3) | 5,486 (5.6) | 8,892 (8.2) |
| Region of birth |  |  |  |  |
| Africa | 4,758 (2.2) | 1,576 (3.0) | 2,018 (2.1) | 2,748 (2.5) |
| The Americas | 5,155 (2.4) | 1,075 (2.1) | 2,443 (2.5) | 2,829 (2.6) |
| Asia or Oceania | 19,559 (9.1) | 5,209 (10.0) | 11,020 (11.2) | 14,518 (13.5) |
| Europe | 17,745 (8.2) | 4,896 (9.4) | 8,098 (8.3) | 9,032 (8.4) |
| Sweden | 167,586 (77.8) | 39,294 (75.3) | 74,245 (75.8) | 78,627 (72.9) |
| Missing | 476 (0.2) | 132 (0.3) | 154 (0.2) | 166 (0.2) |
| Education level ^a^ |  |  |  |  |
| Primary | 19,343 (11.1) | 5,004 (12.9) | 10,580 (12.8) | 10,893 (11.0) |
| Secondary | 59,692 (34.3) | 14,168 (36.4) | 30,440 (36.8) | 37,497 (38.0) |
| Tertiary | 92,052 (52.9) | 18,867 (48.5) | 40,339 (48.8) | 48,845 (49.4) |
| Missing | 2,832 (1.6) | 870 (2.2) | 1,307 (1.6) | 1,569 (1.6) |
| Disposable income quartile ^a^ |  |  |  |  |
| Quartile 1 | 29,336 (16.9) | 7,654 (19.7) | 14,652 (17.7) | 17,101 (17.3) |
| Quartile 2 | 46,511 (26.7) | 10,052 (25.8) | 21,184 (25.6) | 25,900 (26.2) |
| Quartile 3 | 49,356 (28.4) | 10,422 (26.8) | 22,848 (27.6) | 27,644 (28.0) |
| Quartile 4 | 48,228 (27.7) | 10,659 (27.4) | 23,836 (28.8) | 27,990 (28.3) |
| Missing | 488 (0.3) | 122 (0.3) | 146 (0.2) | 169 (0.2) |
| Days with sickness benefit in 2019 ^a^ |  |  |  |  |
| 0 | 156,854 (90.2) | 35,284 (90.7) | 75,143 (90.9) | 89,873 (91.0) |
| 1-30 | 7,363 (4.2) | 1,508 (3.9) | 3,290 (4.0) | 3,893 (3.9) |
| >30 | 9,193 (5.3) | 1,991 (5.1) | 4,074 (4.9) | 4,853 (4.9) |
| Missing | 509 (0.3) | 126 (0.3) | 159 (0.2) | 185 (0.2) |
| Comorbidities |  |  |  |  |
| Asthma | 17,732 (8.2) | 4,253 (8.2) | 7,366 (7.5) | 8,133 (7.5) |
| Cancer | 2,021 (0.9) | 457 (0.9) | 940 (1.0) | 1,415 (1.3) |
| Cardiovascular disease | 18,144 (8.4) | 4,636 (8.9) | 9,635 (9.8) | 14,168 (13.1) |
| Chronic kidney failure | 1,555 (0.7) | 371 (0.7) | 699 (0.7) | 1,220 (1.1) |
| Chronic liver disease | 996 (0.5) | 239 (0.5) | 469 (0.5) | 646 (0.6) |
| Chronic lung disease (not asthma) | 1,782 (0.8) | 487 (0.9) | 880 (0.9) | 1,505 (1.4) |
| Diabetes (type 1 or 2) | 6,164 (2.9) | 1,525 (2.9) | 3,448 (3.5) | 5,076 (4.7) |
| Immunocompromised state | 6,391 (3.0) | 1,578 (3.0) | 2,605 (2.7) | 3,226 (3.0) |
| Mental health disorder | 50,959 (23.7) | 11,109 (21.3) | 21,033 (21.5) | 25,248 (23.4) |
| Neurologic disease | 2,264 (1.1) | 467 (0.9) | 642 (0.7) | 1,739 (1.6) |
| Obesity | 10,170 (4.7) | 2,342 (4.5) | 4,505 (4.6) | 5,442 (5.0) |
| COVID-19 vaccination status before infection ^a^ |  |  |  |  |
| Unvaccinated | 21,757 (12.5) | 13,909 (35.7) | 80,086 (96.9) | 98,698 (99.9) |
| 1 dose | 5,044 (2.9) | 3,402 (8.7) | 2,080 (2.5) | 105 (0.1) |
| 2 doses | 123,289 (70.9) | 21,136 (54.3) | 500 (0.6) | 1 (0.0) |
| 3 doses | 23,829 (13.7) | 462 (1.2) | 0 (0.0) | 0 (0.0) |
| Severity of acute SARS-CoV-2 infection |  |  |  |  |
| Not hospitalized | 214,063 (99.4) | 50,937 (97.6) | 93,968 (95.9) | 103,236 (95.7) |
| Hospitalized | 1,166 (0.5) | 1,136 (2.2) | 3,670 (3.7) | 4,301 (4.0) |
| Admitted to the ICU | 50 (0.0) | 109 (0.2) | 340 (0.3) | 383 (0.4) |

a. Analysis restricted to individuals aged 18 years or older

**Note:** Quantitative variables are presented as median (interquartile range) and categorical variables are presented as count (percentage).

**Abbreviations:** COVID-19=Coronavirus disease 2019

**Figure S2. Cumulative incidence of PCC diagnosis, moving out and death in the study cohorts**

**Note:** The cumulative incidence was calculated with the Aalen and Johansen estimator [10].

**Abbreviation:** PCC=Post COVID-19 condition, SARS-CoV-2=Severe acute respiratory syndrome coronavirus 2

**Supplementary references**

1. Rolfhamre P, Janson A, Arneborn M, Ekdahl K. SmiNet-2: Description of an internet-based surveillance system for communicable diseases in Sweden. Euro Surveill Bull Eur Sur Mal Transm Eur Commun Dis Bull **2006**; 11:15—16.

2. Notifiable diseases - The Public Health Agency of Sweden. Available at: https://www.folkhalsomyndigheten.se/the-public-health-agency-of-sweden/communicable-disease-control/surveillance-of-communicable-diseases/notifiable-diseases/. Accessed 9 March 2023.

3. Swedish Government. Smittskyddslag (2004:168). Available at: https://rkrattsbaser.gov.se/sfst?bet=2004:168. Accessed 29 September 2022.

4. Hergens M-P, Bell M, Haglund P, et al. Risk factors for COVID-19-related death, hospitalization and intensive care: a population-wide study of all inhabitants in Stockholm. Eur J Epidemiol **2022**; 37:157–165.

5. Hedberg P, Granath F, Bruchfeld J, et al. Post COVID‐19 condition diagnosis: A population‐based cohort study of occurrence, associated factors, and healthcare use by severity of acute infection. J Intern Med **2023**; 293:246–258.

6. Statistics Sweden. About Statistics Sweden. Available at: https://www.scb.se/en/About-us/. Accessed 29 September 2022.

7. Public Health Agency of Sweden. Nationella vaccinationsregistret — Folkhälsomyndigheten. Available at: https://www.folkhalsomyndigheten.se/smittskydd-beredskap/vaccinationer/nationella-vaccinationsregistret/. Accessed 9 March 2023.

8. Mårtensson J, Engerström L, Walther S, Grip J, Berggren RK, Larsson E. COVID-19 critical illness in Sweden: characteristics and outcomes at a national population level. Crit Care Resusc **2020**; 22:312–320.

9. Zettersten E, Engerström L, Bell M, et al. Long-term outcome after intensive care for COVID-19: differences between men and women—a nationwide cohort study. Crit Care **2021**; 25:86.

10. Aalen OO, Johansen S. An empirical transition matrix for nonhomogeneous Markov chains based on censored observations. Scandinavian J Stat 1978; 5:141–150.
